# A comparative review of the mineralogical and chemical composition of African major bauxite deposits

**DOI:** 10.1016/j.heliyon.2023.e19070

**Published:** 2023-08-12

**Authors:** N.M. Zainudeen, L. Mohammed, A. Nyamful, D. Adotey, S.K. Osae

**Affiliations:** a Department of Nuclear Sciences and Applications, School of Nuclear and Allied Sciences, University of Ghana, P.O. Box LG 80, Legon, Accra, Ghana; b Institute of Industrial Research, Council for Scientific and Industrial Research, P. O. Box LG 587, Legon, Accra, Ghana; c Department of Nuclear Engineering, School of Nuclear and Allied Sciences, University of Ghana, P.O. Box LG 80, Legon, Accra, Ghana; d Institute of Scientific & Technological Information, Council for Scientific and Industrial Research, P. O. Box CT 2211, Cantonments, Accra, Ghana

**Keywords:** Bauxite ore, African countries, Comparative studies, Mineralogical composition, Geochemical composition

## Abstract

Bauxite, which is the main raw material that aluminium is extracted from was discovered in Africa in the early 1900s. Currently, the production and export capacities of the African Bauxite ore are about a third of the World's total capacity. However, the processes leading to the final finished product of; surface mining of the ore, refining ore into alumina and finally extracting the pure aluminium metal in high energy consuming smelters that employ the Hall-Héroult electrolysis process; seldom take place inside Africa. The main goal of this work is to analyse the mineralogical and geochemical characteristics of bauxite deposits from some prominent bauxite producing and exporting countries of Africa in order to fashion out if a trend exist for the type of source rocks. Judging from the data obtained, gibbsite is found to be the main aluminium oxide in all the bauxite deposits with slight occurrence of boehmite in 3 out of the 13 deposits, while goethite is the main oxyhydroxide iron mineral. The compiled results of the various investigations highlighted the fact that the deposits are of diverse qualities with respect to world standard of major element content of bauxite; with average percentage concentration in the ranges as: Al_2_O_3_ (43.73–61.25), Fe_2_O_3_ (1.55–34.25), SiO_2_ (0.42–10.84); except two of the deposits with alumina content less than 40%. With evaluated silica moduli less than 8 for only two (2) of the deposits (4.76 and 6.94), the rest have higher moduli that ranges between (14.49 and 75.45). The higher percentage of iron oxide content (>20) in six (6) out of the 13 ore deposits, allowed the deposits to be grouped into three (3) categories of grades; high alumina ore, ferruginous ore, siliceous ore and combination of each. Source rock of the deposits were determined through geochemical and petrographic considerations of laterisation products of the rocks through evaluation of the weathering indices of; Chemical Index of Alteration which was in the range (97.16–99.98) while the Ruxton ratio ranged between (0.0133–0.2100); signifying the parent rock underwent intensive weathering process. This is indicative of the source rocks of the Bauxite deposits being either (i) anorthositic, (ii) argillite and dolerite, (iii) granulite and feldspathic gneiss, and/or, (iv) mafic-basaltic andesite igneous. Awareness of new and yet-to-commence emerging bauxite producing African countries was created, by highlighting the economic impact those respective countries will experience when that mining sector is developed for the aluminum industry at home and world at large.

## Highlights

• The top African countries with high bauxite production capacity
• Comparative analysis of the mineralogical and geochemical composition of the bauxite from the top producing African countries
• Emerging Bauxite producing countries of Africa
• Mineralogical composition based on XRD
• Geochemical composition based on XRF and ICP-AES
• Quality of bauxite ore for aluminium production

## 1 Introduction

Aluminum (Al) is the most plentiful metal in earth's crust, representing more than 7% by weight, and is the third most abundant element after silicon and oxygen [1]. Because aluminum is highly reactive, it is mostly found in oxidized form, of which approximately 250 different minerals exist [2,3]. Bauxite is the main source of the world's aluminium, supplying 99% of metallic aluminium. Thus Bauxites are economic concentrations of aluminum, developed from the weathering of aluminosilicate-rich parent rocks [4]. Aluminium in bauxites is known to be precipitated in the form of gibbsite [Al(OH)_3_] or amorphous Al-hydroxides.

As of January 2016, the worldwide bauxite reserves stood at 27.5 billion tonnes. Global bauxite production was estimated at 279.7 million tonnes in 2015, down 0.3% year-over-year. And this was attributed to a production decline during the year in Brazil, Indonesia and China [5]. In 2017, the world bauxite resources were estimated to be 55–75 billion tonnes, comprising Africa (32%), Oceania (23%), South America and the Caribbean (21%), Asia (18%), and elsewhere (6%) [4,6].

### 1.1 Bauxite producing countries in Africa with respective resources

The main Bauxite producing countries in Africa for the sole purpose of alumina production are Guinea, Sierra Leone, Ghana, Mozambique, and to a lesser extent, Tanzania. Other countries, for example Cameroon, do mine bauxite but not mainly for alumina production due to its low quality as a result of high iron content and diminutive interest from mining investors, Tsamo et al. [7] since inadequate information exist on the massive virgin suspected deposits in the Adamawa region, located at the North-eastern part of the country [8]. Other countries such as; Guinea-Bissau, Cote d’Ivoire, Mali and Nigeria have been conducting explorations during the past decade on suspected potential Bauxite deposits [9]. The production capacities and reserves for these African countries can be found in Table 1. Out of these African countries, only Guinea has significantly contributed to worldwide production, with over 80% of African bauxite since 1950 as reported by Knierzinger [10]. However, almost all the bauxite ore gets exported in its raw form as a result of the non-existent or non-functioning refinery on the continent.

**Table 1:** Summary of bauxite resources in Africa.

Country	Estimated Total Reserve (tonnes)	Estimated Mineable Reserve (tonnes)	Estimated Yearly Export (tonnes)	Status of Mining Operations	Reference
Guinea	15,300,000,000	7400,000,000	82,000,000	Operational	[2,6,13,21]
Sierra Leone	104,000,000	–	1,730,000	Operational since 2010 [18]	[[22], [23], [24]]
Ghana	960,000,000	554,000,000	1,400,000	Operational	[2,13,22]
Mozambique	2,000,000	–	6500 (2020) 8,870 (2019)	Operational	[14,22]
Cameroon	550,000,000 (*before 2019*) 892,000,000 (*as at Nov. 2020*)	458,000,000	–	Not yet until late 2023	[11] [12] [25]
Tanzania	–	37,000,000	26,000 (for 2020)	Operational	[26] [27]
Guinea Bissau	17,000,000			Not yet started	[28,29]
Ivory Coast	34,500,000		750,000 (2019) 700,000 (2020) production	Operational since 2018	[30] [31] [32]
Mali	1,630,000,000 (572 million tons of smelter grade)		775,000 (2019 production)	Not Operational	[30] [33]
Nigeria	Mambilla Plateau 1,000,000			Halted since 1999 [10,18]	[34]

### 1.2 Countries with mineralogical and geochemical characterization data of bauxite ore

From the open literature only six countries, namely: Cameroon, Ghana, Guinea, Mozambique, Sierra Leone and Tanzania have got some of their bauxite deposits characterized based on the compositions of the oxides present. Apart from Cameroon which is yet to start mining and exporting of its Bauxite as reported recently in African Business and New African magazines [11,12], the rest are already engaged in mining of their Bauxite ore [10,13]. It is based on this information that this work will seek to ascertain whether the quality of the bauxite is a function of the establishment of the refinery plant at locations closer to where the bauxites are being mined. Though mining has been taking place in Mozambique since 1935, an up to date comprehensive characterization of the ore is not available in the open literature since the last one conducted by Dos Muchangos [14], in 1960.

At Bauxite refineries Engineers modify the Bayer process depending on the number of impurities in the bauxite ore likely to affect the alumina production. These impurities identified mainly as silica, iron oxide and/or oxalate derivatives according to Ref. [15], are an intrinsic part of the ore so have to be removed or minimised in the pre-desilication and desilication processes, before the actual Bayer process commences. In practice, many plants deviate significantly from this general process as a result of desilication [1,15,16], depending on the specific characteristics and chemical compositions of particular bauxite ore feedstock.

### 1.3 Challenges with siting of bauxite refineries on the continent

Worldwide, there are 61 refineries out of which only one (1) is located in Fria, Guinea. This Fria refinery with a capacity of 0.6 million tons, annually processes only about 1% of world output since it started production in 1960 [1,10]. The problem associated with siting a refinery has been the disposal of the bauxite tailings (red mud) that is accumulated during the course of the Bayer process. For example, up until the end of 1980 this red mud in Fria was dumped in the Konkouré River which flows not far from the refinery. The most probable motivation for organizing large-scale mining as in Guinea, without recourse to strict environmental standards, is probably facilitated by the high profitability of production using a cheap labor force and low environmental requirement [6]. The evidence of this perception is the practice of the tailings disposal from the Fria refinery. Though attempts are being made to resolve this problem, the feasibility studies regarding the resolution of this red mud footprint are taking too long. For example to this day, according to Knierzinger [10], people have to cross contaminated streams and cannot use the groundwater for agriculture, bathing, or household needs in the vicinity. Though an ad-hoc solution prescribed in 2012 of closing the refinery is still in force, it cannot be sustained since the production capacity of the Guinean mining is increasing while calls for new refineries to be established on the continent are gaining momentum [1,6,10].

In the absence of proper enforcement of environmental regulations when siting or locating suitable red mud impoundments, modifications of landscapes within catchment areas are likely to occur. The consequences of neglect or delay in upfront feasibility studies on the location of red mud impoundment, according to Ref. [1], could result in: (i) contamination of water resources with caustic soda and metallic oxide-bearing impurities, (ii) direct contact with fauna and flora, (iii) evaporation that could originate highly alkaline rainfalls and (iv) visual impact on extensive areas; since people living in the catchment areas and downstream of the river utilises the river water for their daily activities.

This red mud foot print has become a global concern to an extent that mining companies dealing in bauxite and its processing put a lot of consideration into siting a plant due to envisaged pollution the tailings to be generated could pose. For example, the Bosai Company (China), operating under the Ghana Bauxite Company Limited at Awaso is currently reluctant to build a processing plant in Ghana even though the government and the Chinese partners entered into an agreement to establish a refinery at the appropriate location for the processing of Bauxite since 2010 [17]. The refinery should have been operational in 2014, however, the Chinese reneged on their promise [10,18].

**Fig. 1:**
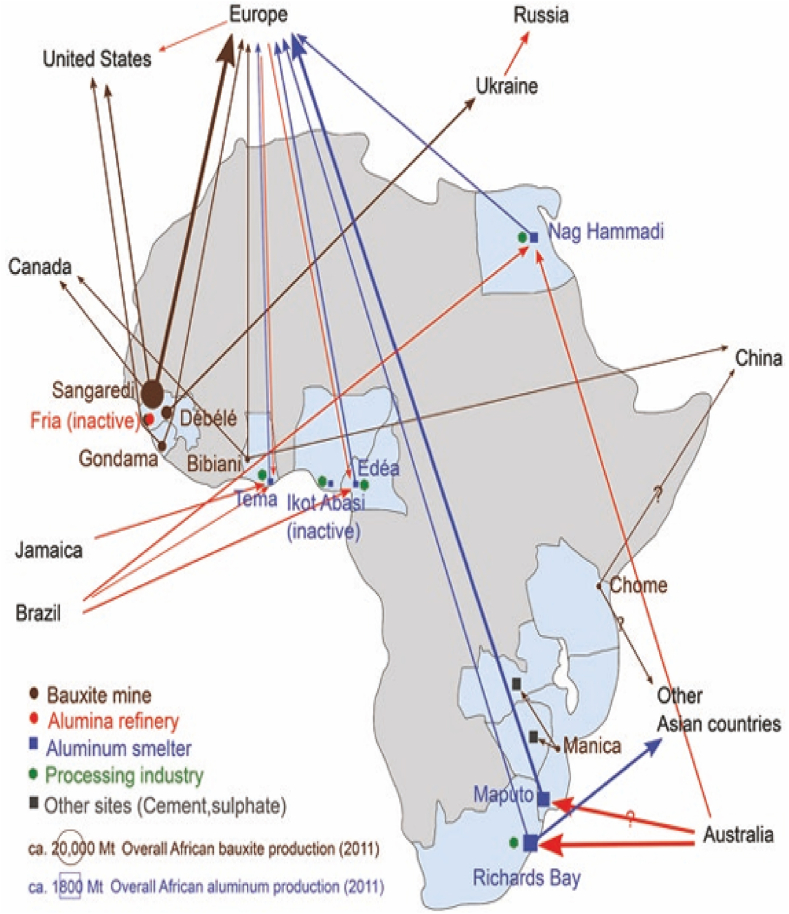
Destination routes: Aluminium, alumina, and bauxite production in Africa.

*Source:* Adapted from [10].

Apart from the over 29% of global output of bauxite from Guinea alone [9], and about 4% from other countries put together [4], Africa plays a significant part in the global aluminium industry mainly by providing cheap energy and labor for smelters in Mozambique, South Africa, Egypt, Cameroon, Nigeria, and Ghana [10]. Cameroun, Nigeria, Tanzania and South Africa also have reserves but as at 2018, no significant data was available for bauxite production and export [10,19,20]. Except for the two small mines in Tanzania and Mozambique, Africa's bauxite is shipped to Europe, the US, and Asia as shown in Fig. 1. In the case where smelters are located, the figure indicates where the alumina is preferably imported from.

In most cases these exported bauxite gets processed into alumina only to be reimported back to Africa in the form of aluminium oxide to feed the smelters dotted around the continent as mentioned earlier. At the smelters where the Hall-Héroult process takes place, the Al_2_O_3_ undergoes an electrochemical reduction process to obtain pure aluminium. Due to the paucity in the factories utilizing aluminium as raw material, the aluminium resulting from the smelters also mainly get shipped out, only to be reimported in the form of aluminium bars, building materials, car parts, and other consumer goods; consequently, aluminium processing in Africa is marginal.

The main objective of this work is to review the various chemical compositions of the representative bauxites from the African bauxite producing countries whose constituents have been determined scientifically. Those statistically treated results that allowed conclusions to be drawn about relations between the major mineral components and chemical composition of the various bauxite deposits considered have been recorded in Table 5, Table 6. Since each refinery, depending on the type of bauxite, operates on unique Bayer process technology [6,15,16] to obtain alumina, it has become necessary to review the chemical characteristics of the ore that are mined in the bauxite producing countries of Africa to ascertain possible reasons for non-siting of refineries. This would be done based on the available information in the literature on the investigated ore deposits; regarding methods of analyses and respective instruments utilized in determining the chemical compositions. By so doing it will become evident why African bauxite is still being processed out, as depicted vividly in Fig. 1, only to be reimported as a finished product for African consumption.

**Table 2:** Bauxites categories according to uses. *Source:* Adapted from [14].

Uses	Metallurgy	Chemistry	Cement	Refractory	Abrasive
Requisites	High alumina	High gibbsite	Moderate or	High alumina	High alumina
	Low iron	Very low iron	high alumina	Low iron	Low iron
	Low silica	Low silica	Low silica	Low silica	Low silica
	Low titania		High iron	Very low alkalis	Low titania
Al_2_O_3_ (%)	>50	>55	45–50	>5 (85)	80–88
SiO_2_ (%)	>5	5–9	<6	<5 (8)	4–8
Fe_2_O_3_ (%)	5–30	1.5–3	>10	<2 (2.5)	2–5
TiO_2_ (%)	0–6	2–4	<3	<3 (4)	2–5
Na_2_O + K_2_O (%)	None	None	None	<0.2	None

**Table 3:** Quality of Bauxite classification based on the literature sources.

τ = Al_2_O_3_/SiO_2_	Classification of bauxite with respect to only Al industry
τ>10	Considered to be of high quality
8<τ<10	Intermediary viable
τ<8	Not economically viable

**Table 4:** Quality of Bauxite classification based on the inequality and regression analysis.

τ = Al_2_O_3_/SiO_2_	Classification of bauxite	Inequality
τ > 20	High alumina ore	τ>20
τ = 11-20	Alumina ore	11≤τ≤20
τ = 4-10	Siliceous ore	4≤τ≤10
τ < 4	High siliceous ore	τ<4

**Table 5:** Mineralogical compositions of the Bauxite ore *(*Tr *= trace amount:* √ = *present: -- = nil)*.

Mineral	Formula	Cameroon				Ghana			Guinea			Mozambique	Sierra Leone	Tanzania
		Min-Mtp 1	Min-Mtp 2	H-Dan	Min-Mtp 3	Awaso 1	Awaso 2	Awaso 3	Balaya Plateau 1	Sangaredi Plateau	Balaya Plateau 2			
Gibbsite	Al(OH)_3_	√	√	√	–	–	–	√	69.5	91.85	83.9	√	√	√
‘Boehmite	γ- AlO–OH	–	–	√	–	–	–	√	–	3.3	–	–	–	√
Goethite	FeO−(OH)	–	–	√	–	–	–	√	–	1.35	4.5	√	–	(Tr)
Anatase	TiO_2_	–	–	√	–	–	–	–	1.74	1.05	1.53	–	√	–
Hematite	$\propto -{Fe}_{2}O_{3}$	–	–		–	–	–	√	23.8	0.7	4.1	–	–	(Tr)
Magnetite	${Fe}_{3}O_{4}$	–	–	√	–	–	–	–	–	–	–	–	–	–
Kaolinite	$H_{4}{Al}_{2}{Si}_{2}O_{9}$p2p${Al}_{2}{Si}_{2}O_{5}{\left(OH\right)}_{4}$	√p2p√	–p2p√	–	–	–	–	√	–	0.4	1.1	√	√	√
Quartz	$SiO_{2}$	–	√	–	–	–	–	√	1.74	0.6	–	–	√	√
Rutile	TiO_2_	–	–	–	–	–	–	–	–	0.6	1.0	–	–	–
Dawsonite	$NaAlCO_{3}{\left(OH\right)}_{2}$	–	–	–	–	–	–	–	–	0.1	–	–	–	–
Diaspore	∝−[AlO]−OH	–	–	–	–	–	–	–	–	0.2	1.3	–	–	–
Goethite, Aluminian	$\propto -\left({Fe}^{+3}Al\right)O\left(OH\right)$	–	–	–	–	–	–	–	–	–	6.15	–	√	–
Maghemite	$\gamma -{Fe}_{2}O_{3}$	–	–	–	–	–	–	–	–	–	–	–	–	√
Mullite	${Al}_{2.25}{Si}_{0.75}O_{4.875}$	√	–	–	–	–	–	–	–	–	–	–	–	–
Anorthite	$Ca{Al}_{2}{Si}_{2}O_{8}$	–	√	–	–	–	–	–	–	–	–	–	–	
Perovskite	$CaTiO_{3}$	–	–	–	–	–	–	√	–	–	–	–	–	–
Sodalite	${Na}_{2}O.{Al}_{2}O_{3}.SiO_{2}$	–	–	–	–	–	–	√	–	–	–	–	–	–
Calcium-aluminium- silicate	${Ca}_{2}{Al}_{2}\left(SiO_{4}\right){\left(OH\right)}_{8}$	–	–	–	–	–	–	√	–	–	–	–	–	–
Zircon	ZrSiO4	–	–	–	–	–	–	–	–	–	–	–	√	–
Ilmenite	FeTiO3	–	–	–	–	–	–	–	–	–	–	–	√	–
Hematite-aluminian	(Fe1-xAlx)2O3	–	–	–	–	–	–	–	–	–	–	–	√	–
Feldspar	$KAl{Si}_{3}O_{8}$ or $NaAl{Si}_{3}O_{8}$	–	–	–	–	–	–	–	–	–	–	–	–	√
Halloysite	${Al}_{2}{Si}_{2}O_{5}{\left(OH\right)}_{4}.nH_{2}O$	–	–	–	–	–	–	–	–	–	–	√	–	–

$Illite=\left(K,H_{3}O\right){\left(Al,Mg,Fe\right)}_{2}{\left(Si,Al\right)}_{4}O{\left[{\left(OH\right)}_{2},\left(H_{2}O\right)\right]}_{10}$ is unique for the bauxite deposit from Mozambique.

**Table 6:** Chemical Compositions of the various Bauxite ores.

Major Constituents	Cameroon (wt.%)				Ghana (wt.%)			Guinea (wt.%)			Mozambique (wt.%)	Sierra Leone (wt.%)	Tanzania (wt.%)
	Min-mtp 1	Min-mtp 2	H-Dan	Min-mtp 3	Awaso 1	Awaso 2	Awaso 3	Balaya Plateau 1	Sangaredi Plateau	Balaya Plateau 2			
Al_2_O_3_	31.69	27.41	43.73	54.87	55.90	45.00	65.15	46.06	61.25	54.69	58.43	48.7	51.59
Fe_2_O_3_	34.25	29.96	24.43	7.17	11.00	25.88	6.99	23.8	3.99	12.25	1.55	20.3	10.65
SiO_2_	0.42	0.54	2.12	2.44	1.2	1.45	2.75	1.74	1.34	0.76	9.00	3.36	10.84
TiO_2_	7.85	4.70	3.54	4.54	1.60	2.30	1.93	1.74	3.91	2.85	0.27	1.53	1.409
CaO	0.45	–	0.03	0.03	<0.01	0.08	0.06	0.06	0.09	0.05	0.02	0.01	0.125
Na_2_O	–	–	<0.01	–	0.09	0.06	1.05	<0.01	0.01	0.05	0.04	–	–
MgO	–	0.01	0.025	0.03	–	0.11	0.08	<0.01	0.02	<0.05	0.27	–	0.081
P_2_O_5_	0.27	0.12	0.246	0.20	–	0.15	0.15	0.06	0.14	0.074	0.01	–	–
SO_3_	0.31	0.26	–	–	–	–	0.13	0.09	–	0.049	–	–	–
K_2_O	0.03	0.02	0.014	–	–	–	0.04	<0.01	0.02	0.022	1.65	–	0.09
MnO	–	–	0.025	0.02	0.02	0.03	0.01	–	0.01	<0.01	0.06	–	–
V_2_O_5_	–	–	–	–	–	0.02	–	–	–	–	–	–	–
Cr_2_O_3_	–	–	0.148	0.09	–	–	–	0.18	0.07	0.073	–	–	–
Mn_2_O_3_	0.05	0.03	–	–	–	–	–	–	–	–	–	–	–
LOI	24.78	–	24.90	29.85	29.70	24.95	23.08	25.60	28.89	29.69	28.62	26.73	25.89
Total	100.1	–	99.21	99.24	99.52	100.03	101.42	99.36	99.74	100.56	99.92	100.00	100.68
**References**	[16]	[7]	[8]	[25]	[57]	[43]	[58,59]	[42]	[21]	[38]	[14]	[24]	[27]

Min-mtp (1, 2, 3), Minim - Martap plateau, Cameroon, H-Dan, Haleo- Danielle plateau, Cameroon.

Awaso (1, 2, 3), Ghana, Balaya (1, 2).

## 2 Geology of african bauxite ore

Bauxite is defined as a naturally occurring, heterogeneous material composed primarily of one or more aluminum hydroxide minerals, plus various mixtures of silica, iron oxide, titania, aluminosilicate, and other impurities in minor or trace amounts [2]. The largest deposits with higher concentrations and economic grade are to be found in tropical belts, between latitudes 300 N and 300 S [35]. It is a type of rock that consists of one or more aluminum hydroxide minerals [36], most notably gibbsite (Al(OH)_3_), boehmite {γ-AlO(OH)} and diaspore {α-AlO(OH)}according to Bardossy & Aleva [37]. Though diaspore has the same general chemical composition as boehmite it is denser and harder. A typical bauxite rock also contains a mixture of goethite {FeO(OH)}, hematite {Fe_2_O_3_}, the clay mineral kaolinite {Al_2_Si_2_O_5_(OH)_4_}, and a minor amount of rutile/anatase {TiO_2_} [1]. Most commonly, Bauxite is a residual or transported constituent of clay deposits at or near the earth's surface. Due to the occurrence models as elaborated above, a completely satisfactory classification does not exist for them [19,20]. However, as reported by Refs. [35,38], classifying bauxite depends less on the host rock but more on its depositional features. Consequently, there are three basic types of bauxitic deposits:

i) lateritic bauxite deposit, produced by in situ tropical weathering and underlain by aluminosilicate or other rocks;
ii) karstic deposit, covers the uneven, karstified surface of limestone and dolomites, and
iii) Sedimentary bauxites, also called Tikhvin-type bauxites are essentially products of proximal redeposition from the accumulation of lateritic bauxite deposits reworked by surficial sedimentation processes. They, overlie unconformably the surface of different aluminosilicate rocks but shows no direct genetic relation with them as it were, because their material was transported from elsewhere.

Even though most of the discovered bauxite deposits worldwide are lateritic in nature, their geochemistry and genetic implications have not been thoroughly studied as against the karstic bauxite deposits [[38], [39], [40]]. Attention therefore would be on the lateritic bauxite ore that characterizes most of the African Bauxite deposits.

One of the most important world lateritic bauxite reserves are the deposits in Africa, located mostly in West Africa. As reported by Sidibe & Yalcin [38] for example, the bauxite ore deposits are located preferentially along each side of the uplift axis of the West-African shield. Out of the six major bauxite producing countries in Africa, only Tanzania is from East Africa while Mozambique is from the Southern Africa region. The rest, from West Africa are; Cameroon, Ghana, Guinea and Sierra Leone. Using the mineralogical and geochemical characteristics, the ore types were compared. Four different Bauxite deposits were analysed for Cameroon while three different sites on the same Awaso mine were considered for Ghana. Two bauxite plateaus were considered for Guinea; the Balaya and Sangaredi plateaux deposits. For the Balaya Plateau, analytical results of two different locations where extensive investigations took place were considered. Details of these selections are listed in Table 5, Table 6 The selection of the sites was based on literature information that were available for each of the country's deposits. From Table 1 it is obvious the quantity of the reserve in Guinea alone far outweighs the combined total amount of deposits from Cameroon, Ghana, Mozambique, Sierra Leone and Tanzania [1,6,10,41].

It was observed that no two samples possess the same characteristics even though dug from the same area from the data of the investigators. This differences in mineralogical compositions are not surprising because they are known to be dependent on the geographical location and depth of the ore from which they were obtained [42,43]. Since high quality bauxite deposits are becoming scarce, it has become necessary for extensive comparative analysis of the mineralogical and chemical composition of deposits; which are available in relatively large quantities that would be profitable for alumina production to be identified and brought to the fore.

### 2.1 Cameroon bauxite ore

**Fig. 2:**
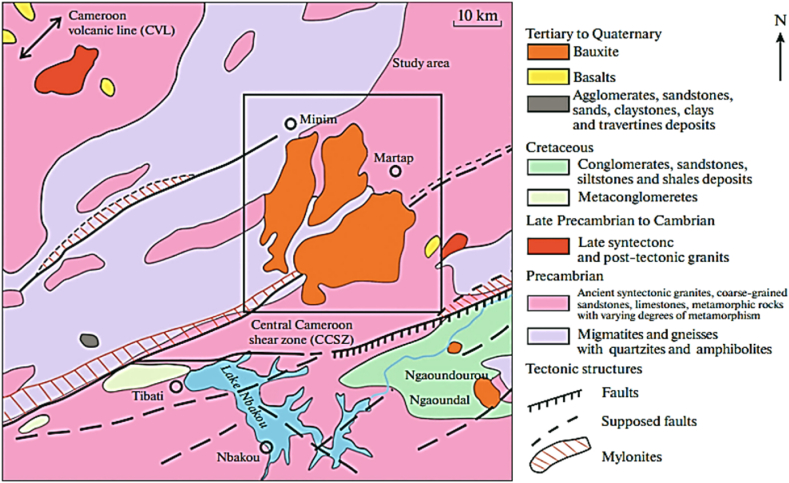
Minim-Martap and Nagaoundal Bauxite deposits on the Danielle plateau in the Vine Division of the Adamawa Region, Cameroon.

*Source:* Adapted and modified from [8].

The bauxite deposit in Cameroon is concentrated within the Adamawa Region, Northeastern Cameroon; where a stretch of about 15 km Bauxite ore plateaus are situated. The total number of the plateaus within the Minim-Martap bauxite district with lateritic bauxite deposits are 11; with the largest being the Haléo-Danielle Plateau deposit [8,25]. This is followed by the Nagaoundal and Minim-Martap deposits; making an overall estimated total of about 2 billion tons [7,16]. Though no mining activity existed before 2020 the information used in this research was based on Laboratory experimentation to ascertain the type of Bayer Process technology that will fit the Bauxite types in anticipation of commercial mining in the future. Currently, it has been reported by Ref. [12], that an Australian firm, Canyon Resources has been given permission and is into bauxite production and export as of 2020. The analytical data utilized here is based on the ore that was collected in the vicinity of Tehabal Aleo with coordinates 06° 53′14″ N and 12°57′55″ E [16] and that at Sabal Haleo on the 06°27′27″ N and 12° 59′ 28″ E [7]. The location as shown in Fig. 2 are all at the Minim-martap plateau deposit; which lies between the Central Cameroon Shear Zone and the Cameroon Volcanic Line; stretching from the Atlantic Ocean in the southwestern part of the country and rises step by step to the east as detailed by Refs. [8,25]. Data sources used in this analyses are from four (4) Bauxite locations in the Mini-martap bauxite plateau district; labelled; Min-mtp 1, Min-mtp 2, Min-mtp 3) and then one (1) located on the Haleo-Danielle plateau (H-Dan).

### 2.2 Ghana bauxite ore

**Fig. 3:**
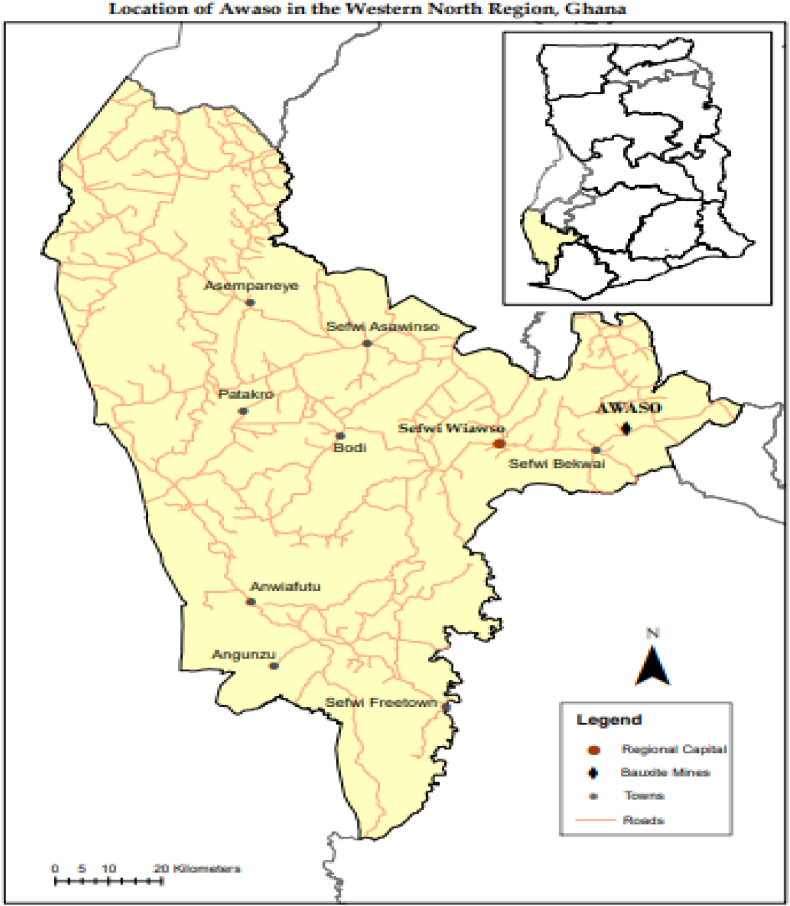
Map of Western-North Region showing the location of Awaso Bauxite mines at Awaso, Ghana.

For Ghana, the location of the study area was the Sefwi-Awaso Bauxite mine in the Western-North Region of Ghana as shown on the map in Fig. 3. The bauxite ore according to Kesse [44,45], rests on a layer of kaolin or lithomarge, which separates it from the underlying slates and the lower Birimian phyllites which strikes at N40E to N80E with steep dips to the NW. This bauxite according to Ref. [45], can be classified as a lateritic silicate type because they were formed as a result of indirect bauxitization processes under tropical weathering conditions. The data obtained from literature were a result of investigations carried out in three (3) different locations within 100 m radius of coordinates 06°13′43″ N and 2° 17′ 21″W [46]. Mining of this bauxite deposit started as early as 1942 and is still ongoing.

**Fig. 4:**
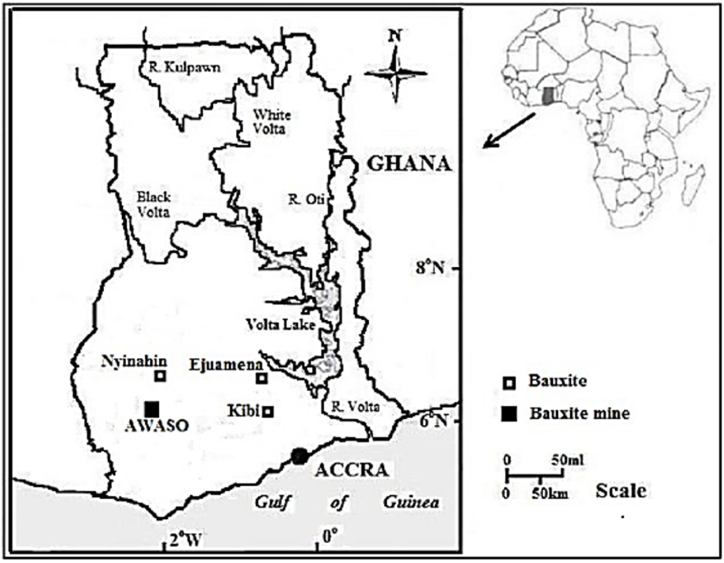
Location of Bauxite deposits in Ghana and Awaso Bauxite mines at Awaso in the Western-North Region, Ghana.

*Source*: Adapted from [17].

There are however other deposits in the country apart from the Awaso mine deposit that are in various stages of prospecting. Information on these prospective deposits is given in section 2.2.1 with their locations illustrated in Fig. 4.

#### 2.2.1 Bauxite deposits in Ghana

Ghana's bauxite deposits are found in Kibi-Atewa, Nyinahin, Awaso and Mountain Ejuanema as illustrated in Fig. 4. Except Awaso which is currently being mined, Nyinahin and Kibi-Atewa are both deposits while Ejuanema is prospect [47]. Of the estimated total bauxite deposits of 2,926 Mt; the Awaso mine has a deposit of about 1,000 Mt; Nyinahin has an estimated 912 Mt; Kibi-Atewa has an estimated reserve of about 200 Mt and Ejuanema has an estimated 814 Mt as reported by Kesse [45]. The bauxite deposits according to Ref. [47] has an estimated percentage alumina (% Al_2_O_3_) compositions of 44.5, 47.7 and 47 for, Nyinahin, Kibi-Atewa and Ejuanema respectively while the respective estimated percentage silica (SiO_2_) composition for Nyinahin, Kibi-Atewa and Ejuanema are 2.5, 3.3 and 4.2. Detailed information on the deposits in terms of mineralogy and geochemistry for the Awaso mine is organised in Table 5, Table 6.

### 2.3 Guinean Bauxite ore

**Fig. 5:**
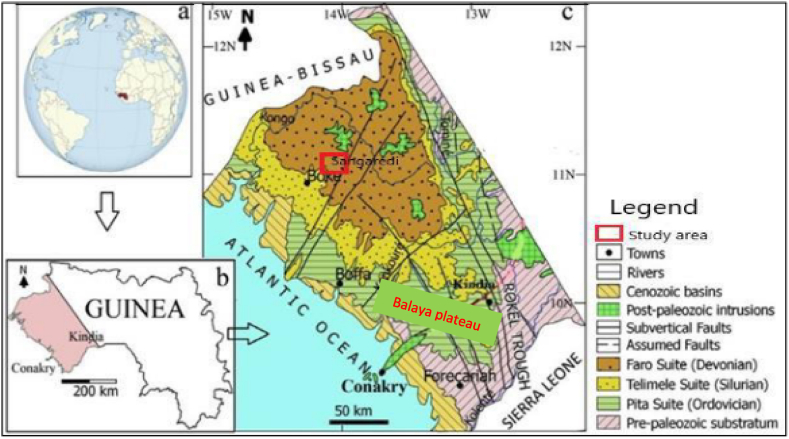
Location of the study areas on the Geologic and tectonic map showing Sangaredi plateau and Balaya plateau and surrounding deposit.

*Source:* Adapted and modified from Ref. [21].

The Republic of Guinea is one of the world's most attractive countries in terms of mining investment in Bauxite. Bauxite was first discovered at the beginning of the 20th century. Currently, it is estimated that Guinea host the largest bauxite reserve in the world, estimated to be about 40 billion tons [1,38], accounting for over 35% of the world's bauxite resources. Guinea has been the second main bauxite exporter after Australia since 2019 [6]. Fig. 5 illustrates the map of the location of Guinean bauxite deposits used in this investigation. While Fig. 5a shows the global location of Guinea as a country, the coloured portion of Fig. 5b delineates the area in the country where concentrations of bauxite is higher as against 5c that details the locations of the specific plateaus with clear boundaries. Hence the information used for this work was obtained from a study carried out on the two Guinean major bauxite locations; at the Balaya plateau and Sangaredi plateau.

i. The Balaya bauxite deposits are formed on the surface of two sub-plateaus; Northern and southern plateaus; laying side by side (herein labelled as Balaya plateau-1 and Balaya plateau-2). The location of this Balaya bauxite plateau is skewed towards the south-western part of the country (Maritime Guinea) in an intracontinental basin which is called Paleozoic Bove Basin; within the south-west part of the West African Craton (WAC) in the Kindia region. The basin according to Sidibe & Yalcin [38], comprises the sedimentary formations of Ordovician, Silurian, and Devonian which are intruded by the Mesozoic magmatic intrusions of the Gondwana breakup of the African platform.
ii. On the other hand, the Sangarédi deposit, located between 11°5′ 23″ N and 13°46'46'' W in the Boké region [21] is the largest bauxite deposit in the world [2,6,13,41]. Most of the bauxite deposits here rest on the weathered schist and sandstone of Devonian intruded by Mesozoic dolerite, which in turn, rest on relatively fresh cordierite hornfels.

### 2.4 Mozambique bauxite deposit

The bauxite ore deposits in Mozambique are located in the northern margin Precambrian greenstone belt in the Manica province. It extends along the Mozambique and Zimbabwe border in the Penhalonga district at Alumen and Marondod at locations; (18° 48^’^ 30 S and 32° 40^’^ 30 E) and (18^0^ 47 50^”^S and 32^0^ 48^’^ 20^”^ E) respectively. This occurrence is within the Precambrian unit of the Manica group, which is built up of Archean and Proterozoic, as well as Phanerozoic magmatic and metamorphic rocks as reported by Dos Muchangos [14].

**Fig. 6:**
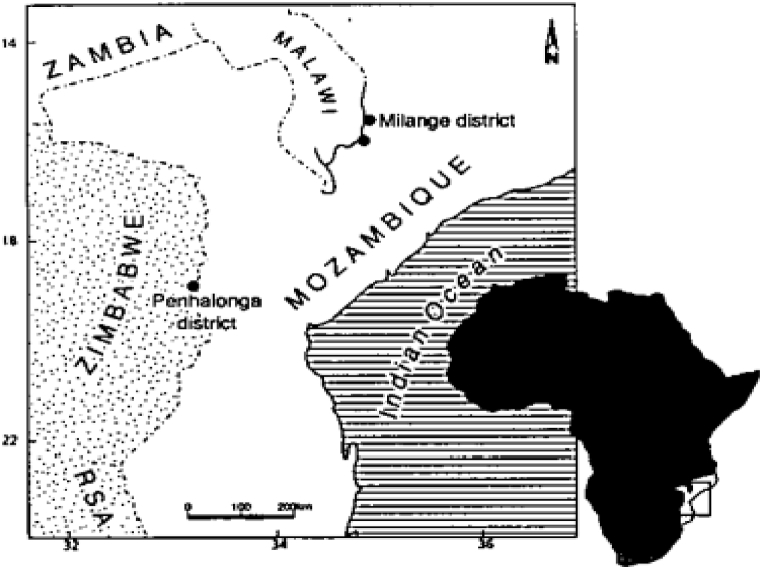
Geographical location of the main areas of Bauxite occurrences in Mozambique: Penhalonga and Milango districts in Southern Africa region.

*Source:* Adapted from [14].

The Penhalonga district is located in the approximately N – S oriented mountain ranges of the northern part of an E – W synclinorium as shown in Figure 6. Production of bauxite from these deposits started in 1935, when four thousand tonnes per annum were mined for the manufacture of aluminium sulphate mainly for water purification industries in Zimbabwe and South Africa [10,14,48]. However, in 2019, Mozambique became the 4th largest bauxite exporter in Africa with 8870 tonnes; following a 20% increase from 2018 production [22].

### 2.5 Sierra Leone bauxite deposits

**Fig. 7:**
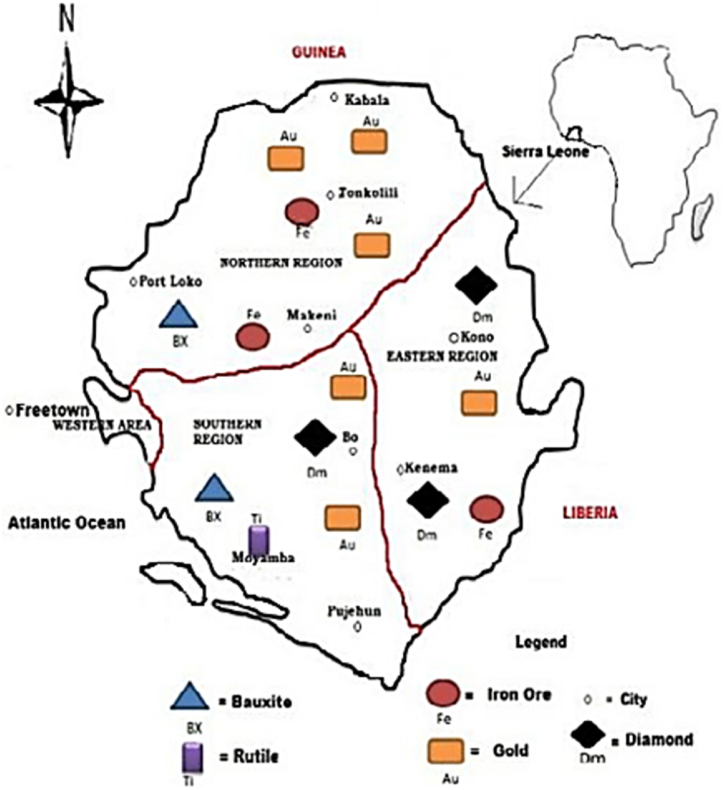
Map of Sierra Leone showing location of Bauxite deposit and other mineral distribution.

*Source:* Adapted and modified from Ref. [23].

The proven bauxite deposit in Sierra Leone is located at three sites; Port Loko (North and South), Kambia and Mamaliki. Out of these, it was only the Port Loko that has been exploited since 1965 [49]. The Bauxite deposits from Sierra Leone were formed from weathering of the hypersthene/feldspar-rich rocks of the Kasila Group under tropical conditions, which resulted in the loss of iron and silica; leaving a high concentration of alumina as reported by Ref. [50]. The Kasila Group is a high-grade metamorphic belt trending NNW, and it forms the coastal rim of the West African Craton. The coordinate location was not provided because the data was obtained from an investigation of the ore that had been exported to Europe [24]. However, the map in Fig. 7 gives a fair idea of the region in the country where Bauxite mining is situated, to be at the North-eastern and South-eastern part. Mining of bauxite started in 1963 [23], until the civil war broke out in 1992 and after the reconciliation, the mining activity resumed in March 2006 [10,23,24].

### 2.6 Tanzania bauxite deposit

**Fig. 8:**
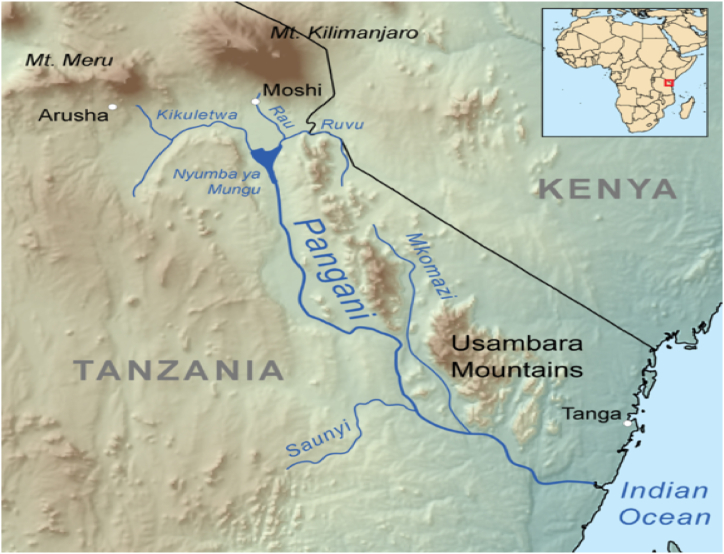
Map of Tanzania showing the location of Usambara Mountains where Bauxite deposits exists, Tonga region, Tanzania.

*Source*: Adapted from Wikimedia commons.

The representative Bauxite deposits from Tanzania whose information was utilized in this investigation are situated in the Usambara Mountains shown in Fig. 8. Located within those mountain ranges on a down-faulted block, which is slightly tilted to the SSE, are two geomorphologically related bauxite plateaus of Mabughai-Mlomboza and Kidundai. The parent rocks of these deposits are mainly granulites and feldspathic gneisses of the Neoproterozoic Mozambique belt. Some of the rock types, which are exposed along with road cuts and in outcrops are made of pyroxene garnet-granulites and hornblende gneisses according to Mutakyahwa et al., [27]; thus these plateaus represent a preserved late Cretaceous–Lower Tertiary old land African surface. Commercial bauxite mining from this deposit started in 2005, and is exported to a cement factory in Zambia [6,10,27].

### 2.7 Quality of ore determination

#### 2.7.1 Classification of bauxite relative to silica and iron content

In order to analyse extensively the quality of the Bauxite deposits with respect to alumina production, two criteria would be followed. Thus, the relative content of iron oxide and silica modulus, (τ); of the deposit as proposed elsewhere [7,8,21,36,51]. Other indicators of the Bauxite quality in relation to uses are given in Table 2. The column on metallurgy and the attributes assigned thereof would be used to assess the quality of the selected ore deposits. Iron oxide constitutes a major impurity in bauxite when the concentration is high (${Fe}_{2}O_{3}>20$) [52]. In order to minimize its effect on the Bayer process, the Bauxite is pretreated so that less reagent is used to dissolve it during the high temperature digestion [43]. Thus with the pretreatment due to iron content, there is a consequential decrease in the amount of waste that could be generated as red mud [15].

**Fig. 9:**
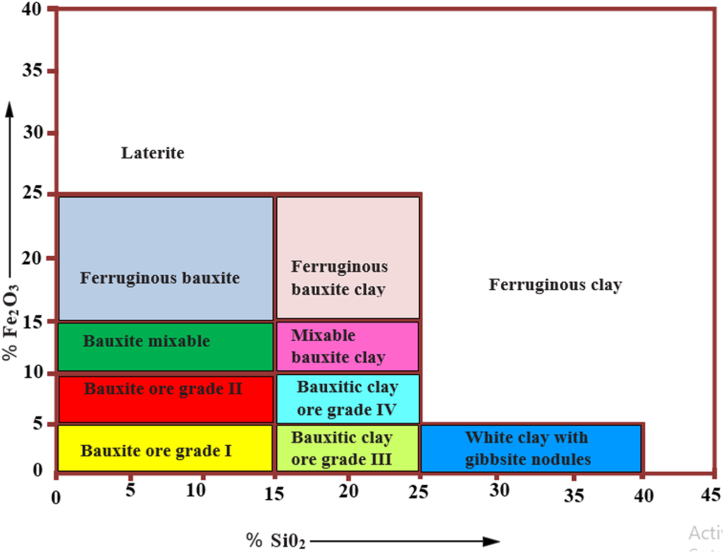
Schematic classification of Bauxite ore deposits, according to percentage content of oxides of silicon (${SiO}_{2}$) and iron (${Fe}_{2}O_{3}$).

*Source*: Adapted with modification from [14].

Fig. 9 is a graphical representation of Bauxite ore classification relative to percentage compositions of the major constitutents; SiO_2_ and Fe_2_O_3_, that was designed by Dos Muchangos [14]. Together with Table 2, Fig. 9 would be used in later analyses of the African Bauxite deposits.

##### 2.7.1.1 Silica modulus

The quality of Ore deposit based on recognized classification, informs the use of a particular ore in industrial applications. Amongst the quality indices for Bauxite ore, are Silica modulus (τ), Ruxton ratio (***R***) and Chemical Index of Alteration (***CIA***) [8,16,21,25,36,51]. While The two indices; ***CIA*** and ***R***; help in determining the source of the parent rock for Bauxite ore; and that would be considered thoroughly in section 2.7.2

Silica modulus by definition is the ratio of the alumina content to silica content of a given bauxite deposit as given in equation (1).

(1) $$\tau =\frac{{Al}_{2}O_{3}}{{SiO}_{2}}$$

A detailed scientific study on the inequality expressions describing the quality of bauxite ore with respect to modulus was undertaken by Yalcin [51]. The deduction of that study summarized in Table 4, was based on the fundamental principle in geology of bauxite where the qualitative oxide contents of the ore allow its identification while the quantitative proportions of these oxides reveal the quality of the ore [25,51].

Careful comparison of Table 3 and Table 4 has revealed that the classification based on, τ , gives similar interpretations with emphasis on the usage of the bauxite relative to aluminium industry only, considering also the economics of the processing as detailed in Table 7. Thus, the desilication stage of Bayer process must be appropriately conditioned during autoclave digestion to obtain a better yield of alumina hydroxide for onward processing to aluminium [15].

**Table 7:** Bauxite quality for Aluminium production.

Country		Silica modulus $\tau =\frac{{Al}_{2}O_{3}}{SiO_{2}}$	Iron content ${Fe}_{2}O_{3}$ wt%	Silica content $SiO_{2}$ wt%	Remarks based on Table 3 [16,21,36]	Remarks based on inequality and regression analysis [51]
Cameroon	Min-mtp 1	75.45	34.25	0.42	Higher (τ), high quality but highly Ferruginous ore	High alumina ore
	Min-mtp 2	50.76	29.96	0.54	Higher (τ), high quality but highly Ferruginous ore	High alumina ore
	H-Dan	20.64	24.43	2.12	Higher (τ), high quality but highly Ferruginous ore	High alumina ore
	Min-mtp 3	22.49	7.17	2.44	Higher (τ), high quality and grade II ore	High alumina ore
Ghana	Awaso 1	46.58	11.00	1.2	Higher (τ), high quality and is a mixable grade ore	High alumina ore
	Awaso 2	31.03	25.88	1.45	Higher (τ), high quality but highly Ferruginous ore	High alumina ore
	Awaso 3	23.69	6.99	2.75	Higher (τ), high quality and grade II ore	High alumina ore
Guinea	Balaya Plateau 1	26.47	23.8	1.74	Higher (τ), high quality but highly Ferruginous ore	High alumina ore
	Sangaredi Plateau	45.71	3.99	1.34	Higher (τ) and grade I ore	High alumina ore
	Balaya Plateau 2	71.96	12.25	0.76	Higher (τ), high quality and mixable grade ore	High alumina ore
Mozambique		6.49	1.55	9.00	Low (τ), not economically viable, and is a grade I ore	Siliceous ore (extra cost in desilication during for Bayer process)
Sierra Leone		14.49	20.3	3.36	Higher (τ), high quality but highly Ferruginous grade ore	Alumina ore (moderate cost relative to desilication in Bayer process)
Tanzania		4.76	10.65	10.84	Low (τ) not economically viable, and is a mixable grade II ore	Siliceous ore (extra cost in desilication during Bayer process)

##### 2.7.1.2 Application of bauxite ore relative to mineral compositions

It was reported [16,21,36,51], that for the application of bauxite in alumina production it is important to determine the silica modulus. From both Table 3, Table 4, it is obvious the higher the silica modulus, the higher the quality of the bauxite as illustrated. Table 3 dwelt on raw quality and economic viability of the ore with no details [36]. However, as indicated by Yalcin [51], there is inverse relationship between the molar compositions of Al_2_O_3_ and SiO_2_ with respect to determination of the modulus using statistical and inequality analyses. Consequently, other categorization was arrived at as shown in Table 4; high alumina, alumina, siliceous and high siliceous ore.

#### 2.7.2 Parent rock determination based on alteration of rock by chemical weathering

As reported by Ref. [8], the major chemical processes of lateritic bauxite formation involves breakdown of the alumino-silicate minerals, the leaching of the alkali and the alkali-earth elements (Na^+^, K^+^, Ca^2+^, and Mg^2+^), and the separation of the Al and Si. The concentrations of these metal ions in the lateritic bauxite products depend immensely on the precursor rock types [4,53]. Therefore both qualitative and quantitative determination of contingent minerals and elemental constituents will assist in the discovery of the type of parent rock. In order to provide a better understanding of elemental mobility due to leaching; and to characterize the weathering profile or degree of chemical alteration that has occurred within the source rock; some measuring instruments were developed. Among those reported are the Chemical Index of Alteration (CIA) [8,21,25,36,54,55] and then the Ruxton Ratio (R) [8,25,54,55].

##### 2.7.2.1 Ruxton Ratio (R)

This is a quotient that results from the ratio of $SiO_{2}$; with relatively high mobility to ${Al}_{2}O_{3}$; which has a low mobility as shown in equation (2). It relates silica loss (leaching) to total elemental loss and considers alumina to be immobile (accumulated) during the weathering.

(2) $$R=\frac{SiO_{2}}{{Al}_{2}O_{3}}$$

When R > 10 means optimum fresh value.

When R = 0 means optimum weathered value.

Although ***R*** resembles the reciprocal of τ, silica modulus, they are both applied differently with respect to characteristics of Bauxite ore deposit. While ***R*** is used to assist in classifying or identifying the source rock of the ore through extent of weathering of rock minerals, its reciprocal, τ, is used to determine the quality of the bauxite ore in relation to uses or applications.

##### 2.7.2.2 Chemical Index of Alteration

By definition,

(3) $$CIA=\left[\frac{{Al}_{2}O_{3}}{{Al}_{2}O_{3}+CaO+{Na}_{2}O+K_{2}O}\right]\times 100$$

The ***CIA*** instrument described by equation (3) was developed by Nesbit & Young [56] to quantitatively evaluate weathering history recorded in sediments and sedimentary rocks. The parameter, CaO in the equation, (3), represent the content in silicate fraction of the ore sample only [8]. ***CIA*** is interpreted as a measure of the extent of feldspars conversion to clays such as kaolinite. Since feldspars are the minerals that dominate the upper crust of laterite, their degeneration via leaching and weathering into laterisation products help in ascertaining the source of parent rock. That determination is based on the fact that Si and Al are major constituents of feldspars; with K being prominent in alkali feldspars while Na and Ca are prominent in Plagioclase feldspars as listed distinctly in Table 5.

Ideally, when.

CIA≤50 minimum fresh value

CIA=100 optimum weathered value

Several interpretations have been given for the different values of the ***R*** and ***CIA*** [54,55]. For example a High CIA means removal of labile cations (Ca^2+^, Na^+^, K^+^ etc) relative to stable residual constituents (Al^3+^, Fe^3+^etc) during weathering processes; suggesting intensive weathering activities. The mineralogical composition of highly weathered rocks is mainly characterized by kaolinite [54]. Thus, a high ***CIA*** imply weathered products are enriched in clay minerals such as kaolinite, smectite or illite with minor feldspars [54,55]. Other categories of intensities of weathering according to Ref. [54] are:

i. High CIA values (97, 95 and 92 on average) means very intensive chemical weathering alteration;
ii. Medium-high CIA values (86, 90 and 92 on average) means moderate-intensive chemical weathering alteration
iii. Medium-low CIA values (between 64 and 70 on average) means moderate-weak chemical weathering alteration and finally
iv. Low CIA values (58 and 55) means weak chemical weathering alteration.

The calculated indices for the various African Bauxite deposits have been listed in Table 8.

**Table 8:** Source rock of Bauxite and its degree of alteration.

Country		Chemical Index of Alteration *CIA*	Remarks based on *CIA* [16,21,36]	Ruxton ratio $R=\frac{SiO_{2}}{{Al}_{2}O_{3}}$	Remarks based on *R* [25,38,54,55]	Source Rock
Cameroon	Min-mtp 1	98.51	High chemical weathering	0.0133	parent rocks were subjected to Less intensive weathering	Lower R and relatively lower CIA means source rock to be alkaline and of Kaolin nature.
	Min-mtp 2	99.93	Extreme high chemical weathering	0.0197	parent rocks were subjected to very intensive weathering	With High CIA value but relatively lower R, the parent rock will probably be iron-rich with source rock being mafic, basaltic andesite igneous
	H-Dan	99.88	Extremely high chemical weathering	0.0485	parent rocks were subjected to very intensive weathering	magnesium-ferrous andesite-basalts (rocks)
	Min-mtp 3	99.95	Extremely high chemical weathering	0.0445	parent rocks were subjected to very intensive weathering	low iron-rich bauxite deposit with source rock being mafic, basaltic andesite igneous
Ghana	Awaso 1	99.82	Extreme high chemical weathering	0.0215	parent rocks were subjected to very intensive weathering	Florencite and xenotime group phases in the lateritic bauxite, hence the parent rock material is of granitic origin
	Awaso 2	99.69	Extremely high chemical weathering	0.0322	parent rocks were subjected to very intensive weathering	With R and CIA comparable to the one above there is possibility that the parent rock though from different location of the same mine, is also of granitic origin
	Awaso 3	98.27	High chemical weathering	0.0422	parent rocks were subjected to Less intensive weathering	With low CIA and relatively higher R, the origin of the parent rock at this location of the mine may not be granitic in nature
Guinea	Balaya Plateau 1	99.83	Extremely high chemical weathering	0.0378	parent rocks were subjected to very intensive weathering	parent rocks are mainly argillites
	Sangaredi Plateau	99.80	Extremely high chemical weathering	0.0219	parent rocks were subjected to very intensive weathering	Various parental rocks, including argillites, dolerites and marine sediments.
	Balaya Plateau 2	99.78	Extremely high chemical weathering	0.0139	parent rocks were subjected to very intensive weathering	parent rocks are argillites
Mozambique	97.16	Moderately high chemical weathering	0.154	Parent rock was subjected to relatively less intensive weathering	Parent rocks are metabasalt and gabbro-anorthositic in nature	
Sierra Leone	99.98	Extremely high chemical weathering	0.069	parent rocks were subjected to intensive weathering	Not enough open literature information to predict	
Tanzania	99.58	Extremely high chemical weathering	0.210	parent rocks were subjected to less intensive weathering	parent rocks for the are mainly granulites and feldspathic gneisses	

## 3 Experimental procedure

### 3.1 Sources of the information and data for the analysis

Information was gathered from open literature and on the internet. Data were extracted and arranged in tabular form for easy comparison of the mineralogical properties of the bauxite at the locations given. Where data from a given location involved different groups of investigators but at different times, a respective numerical figure of that property was taken for each group. For example, all the researchers who investigated Ghanaian bauxite carried out their investigation at Awaso Bauxite mine. So it was those percentage numerical figures of their result for chemical composition of for example, Al_2_O_3_: 55.90, 45.00 and 65.15 respectively for Awaso-1, Awaso-2 and Awaso-3; that were used for the Ghanaian deposits. The same procedure was adopted for the other chemical properties as well and are all organized in Table 6.

No numerical values were explicitly given for the mineralogical properties of the deposits from Cameroon, Ghana, Mozambique, Sierra-Leone, and Tanzania except to quantify as present, existing or in trace amount. However, the investigations carried out on the Guinean Bauxite deposits, resulted in numerical values for the mineral compositions in the ore. For example, Hematite ($\propto -{Fe}_{2}O_{3}$), was captured absent from the Cameroonian and Sierra-Leonean ores but present in the Ghanaian ore and only in trace quantity from Tanzanian ore, as depicted in Table 5. The Hematite from the Guinean ore were however quantified percentage-wise as; 23.8 for Balaya-1, 0.7 for Sangaredi and 4.1 for Balaya-2.

#### 3.1.1 Cameroon

There were five (5) literature sources of information for the Cameroonian Bauxite deposits; one (1) for the Haleo-Danielle plateau and three (3) for the Minim-Martap plateau deposits, all within the long stretch of the Bauxite ore district of Minim-Martap [8,25]. The outcome of the investigations have been reorganized and recorded in Table 5, Table 6 and details could be found elsewhere [7,8,16,25,51].

#### 3.1.2 Ghana

There were five literature sources of information for the current operational Bauxite mine in Ghana, at Awaso. While two (2) of the investigations were carried out at specific locations in the country, one (1) of the results was from Bauxite ore that can be categorized as “composite”, which had been exported from Ghana to Europe [57]. Again the geochemical compositions in numerical terms and the presence or otherwise of the mineralogical compositions have been carefully reorganized and recorded on Table 5, Table 6 respectively. Details of those individual investigations are published elsewhere [17,43,[57], [58], [59]].

#### 3.1.3 Guinea

Investigations carried out on the Bauxite deposits in Guinea has been extensive due to the large reserves it has and the huge interest multinational corporations have for the rich ore [1]. Four (4) main sources of literature information thought to be representative of the wider deposit were used for the Bauxite deposits; two (2) different locations on the Balaya plateau (herein labelled as Balaya Plateau-1 and Balaya plateau-2), all at Debele in the Kindia region of the country and then one (1) on the largest deposit at Sangaredi plateau. Detailed outcome of those investigations can be found elsewhere [1,21,38,42]. Mineralogical constituents and geochemical compositions have been culled and reorganized as shown in Table 5, Table 6

#### 3.1.4 Mozambique

The source of information for the Mozambican Bauxite deposit has been from investigation carried out in the early 1960s [14]. The outcome of those results have been equally reorganized and recorded in Table 5, Table 6.

#### 3.1.5 Sierra Leone

Four different information sources were used for the bauxite deposits in Sierra Leone. While one was done in-situ, the other was from again a composite ore that had been exported from Sierra Leone to Europe [24]. With paucity of information, the available data has been recorded in Table 5, Table 6 Details on the investigations could be found elsewhere [23,24,49,50].

#### 3.1.6 Tanzania

Due to the dearth of information on Bauxite deposits in Tanzania, probably due to its small-scale nature, only the data generated from the explorative investigation of bauxite deposits in Tanzania by Mutakyahwa et al. [27], was used. The raw data were subjected to statistical analysis for each of the chemical properties of mean and standard deviation. Then finally a representative numerical values were chosen for each property. The obtained results have been listed in Table 5, Table 6

## 4 Results and discussion

### 4.1 Petrography (mineralogical analysis)

From Tables 5 and it would be observed that about twenty three mineralogical entities were recorded as being of significant quantities from the generality of the investigations carried out on the Bauxite deposits. Out of the lot, only Gibbsite seems to be common mineral among all the different deposits. The mineral constituents from the Cameroonian deposits in addition are; two different types of kaolinite {Al_2_Si_2_O_5_(OH)_4_ and H_4_Al_2_Si_2_O_9_}, mullite and anorthite [7,16].

The Ghanaian Bauxite deposit on the other hand has Boehmite, goethite, hematite, quartz and rutile as additional minerals [43,58,59]. The uniqueness of this deposit from the others is the presence of perovskite, sodalite and calcium-aluminium-silicate as recorded in the table.

The estimated mineral abundance common to the Balaya-1, Balaya-2 and Sangaredi plateaus Bauxite deposits in Guinea, are Gibbsite, anatase, and hematite, [21,38,42]. While goethite-aluminian occurs exclusively at the Balaya-2 deposit [38], Sangaredi on the other hand has Boehmite and dawsonite exclusively recorded as existing in the ore [21]. While quartz is common to both Sangaredi and Balaya-1 deposits [21,42], Sangaredi and Balaya-2 have in common goethite, kaolinite, rutile and Diaspore as part of their mineral constituents [21,38]. Even though Balaya-1 and Balaya-2 ores; undoubtedly from the same plateau; do not have any mineral constituent in common [38,42], it was concluded from XRD analysis that mineralogical composition of all the Balaya Bauxite deposits are almost identical [38]; a deduction from the fact that Bauxite ores are irregularly scattered in the ore body layer making them show an uneven chemical distribution [8].

Apart from Gibbsite, the deposit from Mozambique showed four more mineral abundance in the form of goethite, kaolinite, Halloysite and Illite [14]. This bauxite; though rich in alumina as shown in Tables 5 and in Table 6 with alumina content to the tune of 58.43%; has not had the benefit of being used for aluminium production but rather as raw material for the production of aluminium sulphate in the water purification industries in Zimbabwe [10,14,48].

Apart from Gibbsite and quartz that runs through all the African Bauxite deposits, the Sierra Leonean deposit has in addition, anatase, kaolinite, goethite-aluminian and quartz. What makes this deposit unique is the presence of Zircon, Ilmenite and Hematite-aluminian minerals in relatively appreciable quantities [24].

The Tanzanian Bauxite deposit has the usual Gibbsite as the common minerals in addition to Boehmite, kaolinite, and quartz in appreciable compositions. There also are present; iron impurities of goethite and hematite but in trace quantities. The uniqueness of this East African deposit is the presence of maghemite and Feldspar in appreciable quantities [27].

### 4.2 Geochemistry

#### 4.2.1 Major elements

The numerical values for the chemical composition are given in Table 6 while the quality of the bauxite deposits with respect to the silica modulus and iron content are given in Table 7.

As oxides, the major elements of the Cameroonian Bauxite deposits are composed of Al_2_O_3_, SiO_2_, Fe_2_O_3_, TiO_2_, and P_2_O_5_ from all the four sites. Except for Min-mtp-3 with Fe_2_O_3_ content of 7.17 wt %, all the deposits from Cameroon have higher Fe_2_O_3_ content; with the compositions from Min-mtp-1 and Min-mtp-2 being higher than that of the Al_2_O_3,_ making them generally ferruginous in nature. The alkali and alkali earth elements (CaO, K_2_O, and MgO) are what occur in the deposits with compositions in the range (0.01–0.45) wt. %. Though the ore deposits have high silica moduli, (τ), ranging between (20.64–75.45) as shown in Tables 7 and it would experience some economic challenge due to the large requirement of reagent to pretreat the Fe_2_O_3_ content before being subjected to the Bayer process [15,43].

The major elements from the Awaso deposits, Ghana that are common to all three deposits as shown in Table 6, are Al_2_O_3_, SiO_2_, Fe_2_O_3_, TiO_2_, CaO and MnO. While Awaso-2 is the only one with recorded V_2_O_5_ and no K_2_O and SO_3_, Awaso-3 has on record all the alkali and alkali earth elements (Na_2_O, K_2_O, and MgO) with compositions ranging between (0.02–1.05) wt. %. With high silica moduli in the range (23.69–46.58) and Fe_2_O_3_ content relatively low, except for Awaso-2 deposit with as high as 25.88 wt %; the generality of the Awaso deposit could be classified as high alumina-grade ferruginous bauxite, since the average of the Fe_2_O_3_ content being 14.64 wt % satisfies that class based on deductions from Fig. 9 and Table 7.

As shown in Table 6, the major elemental oxides of the Guinean ore are Al_2_O_3_, SiO_2_, Fe_2_O_3_, and TiO_2._ The analytical values of these elements from the Balaya plateau-1, Balaya plateau-2 and the Sangarédi plateau have little variation except for the iron content which are 23.80 wt %, 12.25 wt % and 3.99 wt % respectively. The range of values for percentage compositions for the alkali and alkali earth elements are CaO (0.01–0.45), Na_2_O (0.01–1.05), K_2_O (0.01–0.04), and MgO (0.01–0.11).

The composition of P_2_O_5_ in the deposits range between (0.01–0.27); with the deposits from Cameroon recording relatively larger values. This must be of concern to process engineers because accumulation of P_2_O_5_ during the cyclic Bayer process could contaminate the produced alumina especially if it would be used as reduction cell feed [60]. SO_3_ presence is recorded in two deposits from Cameroon and Guinea and one deposit from Ghana. Since the percentage compositions are less than 0.4%, the negative effects of the presence of sulfur as mentioned in Ref. [61] would not arise for all the investigated deposits. MnO, Mn_2_O_3_ and Cr_2_O_3_ occurrence are relatively low and range between (0.01 and 0.18) wt%. MnO is recorded for deposits from Cameroon, Ghana, Guinea and Mozambique. While Cr_2_O_3_ was present for deposits from Cameroon and Guinea, Mn_2_O_3_ was recorded for Cameroon deposits of Mim-mtp-1 and Min-mtp-2.

Based on the higher silica modulus however, the deposits from Guinea can be classified generally as high alumina grade with additional qualification for the Balaya Plateau-1 deposit as ferruginous ore and Balaya plateau-2 as mixable ore in accordance with the contents of Fig. 9 and Table 7.

The oxides of the major elements of the deposit from Mozambique, according to Ref. [14], are Al_2_O_3_, SiO_2_, Fe_2_O_3_, and TiO_2_, while the alkalis and alkali earth elements are (CaO, Na_2_O, K_2_O, and MgO). Though low in iron content of 1.55 wt % and rich in alumina to the tune of 58.43 wt %, it has a low silica modulus (τ) of 6.49. This makes the deposit a siliceous ore as given in Table 7. The low silica modulus imply extra cost in desilication during Bayer process for aluminium production. The low amount of the reserves shown in Table 1, coupled with this low τ could be the underlying factors this Bauxite deposit has not been used for the production of aluminium, but rather exported to Zimbabwe and South Africa for the production of aluminium sulphate in the water purification industries [10,14,48].

The major elements of the Sierra Leonean deposit are composed of Al_2_O_3_, SiO_2_, Fe_2_O_3_, and TiO_2_. There was no record of any alkali and alkali earth elements except CaO which has a concentration of less than 0.01 [24]. Due to the moderately high iron content (Fe_2_O_3_ = 20.3%), this ore could be classified as a medium grade ferruginous bauxite. With about 49 wt % alumina and silica modulus of magnitude 14.49 this ore is economically viable [15]. Except for this high Fe_2_O_3_ content which makes it require pretreatment to lower the total iron content before being subjected to the Bayer process [15,43], this deposit could fit into alumina refineries everywhere in the world.

The major elements of the Tanzanian ore, according to Ref. [27] are Al_2_O_3_, SiO_2_, Fe_2_O_3_ and TiO_2_. The alkali and alkali earth elements detected were CaO, K_2_O and MgO. This deposit, though smaller in quantity as evidenced in Table 1, has a relatively higher alumina content (Al_2_O_3_ = 51.51 wt %) but low silica modulus of magnitude 4.76. With the Fe_2_O_3_ content of 10.65 wt %; almost half that of the Sierra Leonean deposit; this East-African deposit was not expected to be economically viable due to the extra cost needed for desilication during Bayer processing, for high siliceous bauxite ores [15,16,51]. The export of this Tanzanian bauxite ore to Zambia for cement production since mining started in 2005 could be due to the aforementioned characteristics [10].

Due to the craving for value addition to the ore deposits with respect to especially, essential elements (Critical Raw Materials-CRM) that are normally in trace quantities, the presence of characteristic elements in the ore which intervene in the CRM recovery do inform the type of value addition each African country could focus its research on. For example, during Bayer process, vanadium interferes in the industrial extraction of gallium by being co-extracted; apart from its tendency to contaminate the alumina with consequential reduction in electrical conductivity of the aluminium metal.

#### 4.2.2 Trace elements

The trace element components, with their respective concentrations for the most abundant ones, of the Cameroonian deposits at Min-mtp-3 are; Zr (414.35 ppm), Ce (311.47 ppm), Sr (235.68 ppm), V (235.12 ppm), Ba (185.47 ppm), La (171.88 ppm), Nd (166.92 ppm), Ga (58.54 ppm), and Nb (55.76 ppm) [25]. Recorded trace and rare element for deposits from Haleo-Danielle were given as; Zr (667.25 ppm), V (446.4 ppm), Ce (107.93 ppm), Sr (98.46 ppm), Nb (92.1 ppm), La (58.05 ppm), Ga (55.3 ppm), Ba (53.53 ppm), Nd (37.96 ppm) [8]. No trace element determination was available for Min-mtp 1 and Min-mtp-2 [7,16].

There were records of trace elements from the Awaso-3 deposit as established by Ref. [58] but no concentration margins were given. They are; V, Cr, Co, Ni, Cu, Zn, Ga, As, Y, Ba, and Pb. However, Vind & Alexandri et al. [62], reported a concentration of $57\frac{mg}{kg}$ for Ga in Awaso −1 deposit. The numerical value of concentrations of the remaining trace and rare elements for Awaso-1 was detailed by Vind & Malfliet et al. [57], as follows: La (19.1ppm), Ce (34.0 ppm), Nd (13.0 ppm), Sm (2.0 ppm), Eu (0.8 ppm) Yb (2.5 ppm), Lu (0.4 ppm) and Th (22.7 ppm). No data for trace element detection was given for Awaso- 2. Nevertheless, with the relatively sizable concentration for gallium; a CRM; implementation of appropriate extraction technique, could pave the way for the Ga recovery in order to add value to the Ghanaian bauxite deposit.

The information obtained of the trace elements for the Guinean Bauxite deposit from the Balaya plateau-2 bauxite showed high values of Ti, Cr, Ga, Nb, Th, V, Zr, Y, La, Ce, and Nd, with concentrations ranging between 10 ppm and 22200 ppm [38]. For example the concentration of Ga was in the range, 50.9–90.3 ppm. This means that with appropriate technology, these Critical Raw Materials (CRM) could be extracted as well in order to add value to the bauxite ore. However, since more than 50 trace element exist in given bauxites deposits [20,38], it is likely other essential elements do exist but could not be registered because their quantities are negligible and far below the detection limit of the analytical instruments employed [27].

Sangaredi deposit on the other hand, have a relatively low occurrence of the trace elements compared to the Balaya deposits. For example the concentrations ranges, according to Ref. [21], between 13 ppm and 990 ppm. Those recorded trace elements are Sc, Ga, Nb, Sr, Th, V, Zr, Y, La, and Ce. The concentration of Ga was in the range 13 to 65 ppm, a little lower than those from Balaya plateau. While Sc and Sr were exclusively detected at Sangaredi plateau, Ti, Cr, and Nd were exclusively detected in the Balaya plateau deposit [21,38].

While the trace elements found for the Mozambican deposit were given as: Cr, Ni, Co, V, Cu, Zn, Ba, Be, Sr, Li, Zr, Y, Ga, Nb, Th, and U [14], no data was available for trace elements from Sierra Leonean deposit [23,24,50].

According to Mutakyahwa et al. [27], the trace elements that occur in the Tanzanian bauxite ore deposit in considerable amounts were Cr, u, Ni, V, Co, Nb, and Zr.

#### 4.2.3 Source rock of the various bauxite deposits from the selected African countries

According to Patterson et al. [19], when conditions are suitable for weathering, almost all types of rock containing aluminium can give rise to bauxites. This makes it difficult to determine the origin of a given bauxite deposit. However, with indicators like, ***R*** and ***CIA***, which are linked to processes that take place during the laterisation of Bauxite, the source rock determination is narrowed.

It was reported that, when silica in the source rock is quickly dissolved and leached resulting in low ***R*** values in the weathering product, the possibility of aluminium reacting with it to form Kaolinite is very slim [8]. This infers that the precursor rock endured intense laterisation in course of massive chemical weathering.

Except for Mozambique and Tanzania with respective compositions of SiO_2_ at 9.0 wt % and 10.85 wt %, all the silica contents in the bauxite deposits under consideration fall within the range of 0.42 and 3.36 wt %. This means all the ore deposits from Cameroon, Ghana, Guinea, Sierra Leone were devoid of reactive silica and kaolinite; as is realized when the silica content of the ore exceeds 12.0 wt % [8].

From Tables 8 and it could be observed that, all the bauxite deposits experienced intense chemical weathering with ***CIA*** values ranging from 97.16 to 99. 98. The deposit from Mozambique have the least with 97.16, followed by Awaso-3 with 98.27 and Min-mtp-1 with 98.51. The rest of the deposits have ***CIA*** values, in ascending order; of 99. 58 for Tanzania, 99.69 for Awaso-2, 99.78 for Balaya-2, 99.80 for Sangaredi, 99.82 for Awaso-1, 99.83 for Balaya-1, 99.88 for H-Dan, 99.93 for Min-mtp-2, 99.95 for Min-mtp-3 and 99.98-Sierra Leone. When the ***CIA*** value is relatively higher, that translates to less alkaline metals in the respective ore; thus the source rock has undergone intense weathering that resulted in more leaching of the alkaline metals within the laterisation product [8,21,25,36,54,55].

The ***R*** values however show different order; with values ranging from 0.0133 up to 0.210 for Min-mtp-1 as the least and Tanzania the highest. The rest of the ***R*** values are: 0.0139, 0.0197, 0.0215, 0.0219, 0.0322, 0.0378, 0.0422, 0.0445, 0.0485, 0.069 and 0.154 for Balaya-2, Min-mtp-2, Awaso-1, Sangaredi, Awaso-2, Balaya-1, Awaso-3, Min-mtp-3, H-Dan, Sierra Leone, and Mozambique respectively. Higher value of Ruxton Ratio (***R***), translates to the fact that SiO_2_ was leached less during the weathering of the source rock so more of it remained in the weathering product; the Bauxite ore [8,25,54,55].

### 4.3 Non-active bauxite exporting countries

The countries with potential to produce and export their Bauxite deposits are Guinea Bissau, Cote d’Ivoire and Mali. Their deposits are still being discovered while infrastructural developments relative to operation of economically viable bauxite mines is being undertaken.

#### 4.3.1 Guinea Bissau

The survey and prospecting for Bauxite in Guinea Bissau was conducted by the Dutch in the 1950s, and reported an estimated deposit of 120 million tons out of which 24 million tons, presumably the best grade, contained 47% Al_2_O_3_ and 3% SiO_2_ [19,29]. The occurrence of these deposits are at Boe in the south-eastern part of Gabu region of Guinea Bissau, where highly weathered ferrallitic mantles occur within different geomorphological levels [19,63]. The Bauxite deposit location stretches between (11° 40' - 12° 0′) and (13° 40′- 14° 15′), W [63].

Even though the bauxite deposits rest on dolerite sills that overlie impure sandstone, the detected minerals were however; boehmite, gibbsite, goethite, hematite, anatase, quartz and kaolinite [63]. The percentage compositions of the major elements in oxide form are; Al_2_O_3_ (46.5–53.82), SiO_2_ (0.89–3.5), Fe_2_O_3_ (14.87–16.40), and TiO_2_ (1.68–1.79) [19,29]; with additionally reported white bauxite containing 63.42% of Al_2_O_3_ [19].

The bauxite deposits of Guinea-Bissau were considered to be sub-economic because of ecological sensitivity of the surrounding Boe National Park [64]; and of the economic uncertainties about mining it in relation to the underdeveloped nature of the region, where the impoverished Buba port is located. Thus the cost of road haulage and shipping could probably drive up the overall production cost of the ore. However, with the current increase in the bauxite prices at ($49.00 - $61.00) per ton as reported in 2022 [65], Guinea-Bissau's bauxite deposits look significantly more attractive with interest from even Angola indicating its willingness to invest in the road and the port development, as well as the mining infrastructure as contained in Ref. [29], and recently in 2020, reported online [28].

#### 4.3.2 Cote d‘Ivoire

Bauxite deposits in Cote d’Ivoire have been discovered on isolated plateaus at many localities in districts that include Bongouanou, Toumodi, Divo, Sassandra-Lakota, Benene and Tabou [19]. The deposits are mainly gibbsite, with minor quantities of boehmite. With respect to aluminium production, best grade and largest deposits are in the eastern part of the country at Benene district with an estimated deposit of 10.7 million tons that is 50% Al_2_O_3_ as at 1971 [19]. However, an updated estimate of this Benene deposit stands at 35 million tonnes and due to the already functioning infrastructure, the mine went into operation in 2018 as the first of its kind in the Cote d’Ivoire as reported by Bloomberg news, and other online aluminium circles news [18,31,32,66]. With massive investment of nearly $375 million, the mine was able to function such that the first shipment of the ore out of the country to China took place in the second quarter of 2020. Information on the laterisation process of the parent rock with respect to weathering is scarce making it challenging to predict the type of source rock.

#### 4.3.3 Mali

The locations of proven Bauxite deposits in Mali are Torolo (12° 47′ 16″ N; 8° 51′ 5″ W), Falea (12° 16′ 0″ N; 11° 16′ 60″ W) and Bouala (13° 16′ 0″ N; 5° 44’ 0” W) [33,67,68] from two (2) out of the (4) suspected deposits in western regions of the country at; Kayes, Koulikoro, Segou and Sikasso regions; with an estimated 820 million tons of the deposits containing over 40% Al_2_O_3_ content, and less than 4% SiO_2_ [19]. This qualifies the ore of being of world class standard for alumina-bound Bauxite ore which has composition of the major oxides being Al_2_O_3_ > 45%; Fe_2_O_3_ < 20%; and SiO_2_ < 5% [52].

Since Mali is part of the West African, Fouta Djallon-Mandingo bauxite province, it could be assumed that the major mineral components of the deposits are probably boehmite, gibbsite, goethite, and kaolinite [69]. After the last exploration and prospecting in 1950, the total potential bauxite resources in Mali were estimated to be 1 billion tons, with the total reserves estimated to be 880 million tons. In spite of this, no serious mining took place because the deposits were considered to be sub-economic; due to its remote location as the road haulage for long distances to ports for shipment abroad could shoot up the cost of production.

The recent exploration by the Central African mining and exploration company (CAMEC) however, put the Bauxite reserves in Mali to an estimated value of 1.63 billion tonnes, which is equivalent to 572 tonnes of refined aluminium as reported by Mining review.com in 2009 and corroborated by Reuters news in 2017 [33,67]. And due to the infrastructural developments that was carried out by the government, Mali was able to produce an estimated Bauxite ore of 775, 000 tons, a decade after the CAMEC bauxite discovery [30].

## 5 Conclusion

Of the six bauxite producing countries in Africa, only the deposits from Mozambique and Tanzania are utilized completely within Africa. While Mozambique exports its resource to water purification industries in South Africa and Zimbabwe for the production of aluminium sulphate, Tanzania on the other hand, export its bauxite to a cement factory in Zambia. The four remaining countries; Cameroon, Ghana, Guinea and Sierra Leone; with larger deposits, in contrast, export theirs outside Africa to Europe and other parts of the world for sole purposes of alumina and metallic aluminium production.

The respective African bauxite deposits have some similarities. Apart from being lateritic in nature, they all have gibbsite; an indicator of presence of alumina; as the common mineral with larger percentage composition. The relatively smaller percentage composition of quartz has resulted in making the African deposit economical for the respective countries. This is evidenced from the values of the silica moduli that are greater than 10, save for those that are utilized within Africa and not for metallic aluminium production. With this envious quality however, there is still no functioning bauxite processing plant on the continent; a situation that needs examination in order to bring down cost of production for aluminium against the incurred extra charges when the ore is being exported.

The bauxite deposits chemically have all the four major elements of Al_2_O_3_, SiO_2_, Fe_2_O_3_, and TiO_2_ in addition to K_2_O, Na_2_O, CaO and MgO as the alkali and alkaline earth metals. Except for the Cameroonian deposit whose percentage iron concentrations are higher; in some case more than the alumina content save for one deposit on the Min-mtp-3; the rest of the deposits have alumina content far higher than that of Fe_2_O_3_. Thus in the process of refining these other bauxites, less reagent of the soda would be required in the Bayer process with a consequential production of a relatively small quantity of red mud.

The general lateritic classification of the deposits is due to the presence of the alkali and alkali earth metals. And using the quality determinants, the deposits were put into three (3) categories of grades; high alumina ore, ferruginous ore, siliceous ore and combination of each. From the weathering indices that relate amounts of the alkali and alkaline earth metals in the bauxite deposits to the alumina content of same, it was concluded that the parent rocks underwent intensive weathering process; with the nature of the various parent rocks of the deposits being either; (i) anorthositic, (ii) argillite and dolerite, (iii) granulite and feldspathic gneiss, and/or finally, (iv) mafic-basaltic andesite igneous.

Of the three major sources of gallium, Bauxite contributes about 50% and that is even obtained during the refining process of the bauxite ore. So countries with non-negligible concentration of Ga, needs to undertake thorough investigation into the techniques for recovering Ga both in the Bayer liquor and the red mud in order to add more value to the deposits. For example Ghana, with comparably high Ga concentration in its Awaso deposit, need to carry out research into the techniques of gallium recovery in order to add value to its only operational Bauxite mining deposit.

African Bauxite producing countries continue to mine and export bauxite for the conversion into market ready products. And with the number increasing as a result of new discoveries, conscious effort need to be made in setting up Bauxite refinery to ameliorate the challenges encountered during transportation of the ore to ports for onward shipment to other parts of the world.

## Author contribution statement

All authors listed have significantly contributed to the development and the writing of this article. Data availability statement Data will be made available on request.

## Funding

This research did not receive any specific grant from funding agencies in the public, commercial, or not-for-profit sectors.

## Additional information

No additional information is available for this paper.

## Declaration of competing interest

The authors declare that they have no known competing financial interests or personal relationships that could have appeared to influence the work reported in this paper.
